# Paediatric flat foot and foot dimension in Central Anatolia

**DOI:** 10.1186/s12887-021-02645-9

**Published:** 2021-04-27

**Authors:** Serap Alsancak, Senem Guner, Enver Güven, Ali Koray Özgün, Yunis Akkaş, Neslihan Alkıs

**Affiliations:** 1 Department of Prosthetics & Orthotics, Faculty of Health Science, Ankara University, Ankara, Turkey; 2 Department of Anesthesiology and Reanimation, Faculty of Medicine, Ankara University, Ankara, Turkey

**Keywords:** Children, Foot anthropometric, Pes Planus, Pes Cavus

## Abstract

**Background:**

Information on the foot structures of Central Anatolian children is limited. Foot structures of children aged 6–10 years were shown to be different according to sex and increasing age.

**Objective:**

This study aimed to compare the foot anthropometric values by age and sex and collect the foot anthropometric data to reveal the relationship between pes planus and pes cavus in the arches of children according to age.

**Methods:**

Footprints of 335 children (180 boys and 155 girls) aged 6–10 years were taken by the pedigraph method and evaluated using 18 different parameters. The TFL (Truncated foot length), FL (foot length), Arch Index, Chippaux Smirak Index, Staheli Arc Index, and foot rotation values of the children were examined. To examine the relationship between the parameters, normality values were examined. Independent samples t-test was used to analyze sex differences in terms of foot size and shape.

**Results:**

Correlations between other parameters were determined using the correlations analysis method. TFL, metatarsal circumference, and FL were strongly correlated with age in the children. Foot rotation increased with body mass index in the girls compared to that in the boys. According to the evaluation results with the classification made with the Staheli arch index, 63.3% pes planus, 9.8% pes cavus and 27.7% of the normal arch structure were identified.

**Conclusions:**

Planning shoe production accordingly will contribute to the development of healthy feet in children. This article focused on foot structures of in Central Anatolia and to identify early foot deformities in children. This study found that the length of the TFL was smaller in boys than in girls.

## Introduction

Paediatric pes planus is described as decreasing of the medial longitudinal arch and has been researched distribution around the world for years [1]. Pes planus generally has two classification as flexible and fixed pes planus. Flexible pes planus becomes symptomatic or asymptomatic. Most of the symptomatic pes planus causes pain in the feet, problems in the proximal joints, and early fatigue during gait and decreases the quality of life [2–4]. Hindfoot can cause valgus position of flat foot. Pes planus is common in paediatric group and foot arches usually develop with age [5, 6]. Paediaetric flexible pes planus is common, affecting around 48 to 77.9% children [7, 8].

Pes cavus is defined as increasing in height of the medial longitudinal arch. The deformity does not corrected on weight bearing. Elevation of the medial longitudinal arch, forefoot pronation, valgus and adduction, clawing of the toes are typical in this deformity. Hindfoot is varus position and the foot most common causes pes cavovarus. The underlying cause of pes cavus is mostly neurological [9, 10].

When designing shoes for any age group, anthropometric data of that group is of great importance. The use of correct foot sizes improves one’s foot health and comfort. Shoes that do not fit the foot structure used at a young age can lead to permanent foot deformities. However, foot deformities that can be noticed at a young age can be eliminated before reaching the developmental period. Foot measurements can be taken by different methods. These are measuring with a caliper or tape measure, three-dimensional scanning, photographing, or foot-printing [11]. Foot prints are most used method evaluation foot morphology and footprint based measures of children’s feet may represent developmental pes planus, pes cavus and regular foot. The main instruments used to obtain footprints are ink imprints, optical podoscopes, baropodometry, pedography, digital photography, radiography, and platinum scanners. Three measurements are used for footprint diagnosis: Clarke’s angle, the Staheli index and the Chippaux-Smirak index [12]. This study focus on 6–10 years children and evaluate the three most commonly used footprint analysis methods for pes planus, pes cavus: the angle –based Staheli arch index, Arch Index and Chippaux Smirak Index.

When studies on a similar subject are examined, foot morphologies may vary between sexes and different populations. As a result, the data obtained in this study vary according to age and sex [13–15]. Using these data, designing shoes, which is unique to foot morphology, can be beneficial for children’s foot health.

Previous studies have determined the morphological dimensions of feet according to age groups in different nations. In Anatolia, there are studies from anthropological studies such as sex prediction made from adult foot structure when examined. In this study, we aimed to determine the foot dimensions of the 6–10-year-old children in Central Anatolia and to identify early foot deformities in children.

## Materials and methods

This study included a sample group of 335 children (155 girls, 180 boys) aged between 6 and 10 years. The sample group consisted of children living in Central Anatolia, without any walking disorder and foot pathology. Approval was obtained from the ethical board committee of Ankara University of Medical Sciences Ethics Board, and informed consents were obtained from the families of the children included in the sampling before initiation of the study. Parents and children provided their written consent to participate.

Data were collected from 24 different measurements including the shoe size of the dominant foot, metatarsal circumference, TFL, and Staheli arch index. In addition, demographic data such as height, body weight, and body mass index of children were measured. The shoe types and sizes used by the children in the sample were subsequently determined.

**Fig. 1:**
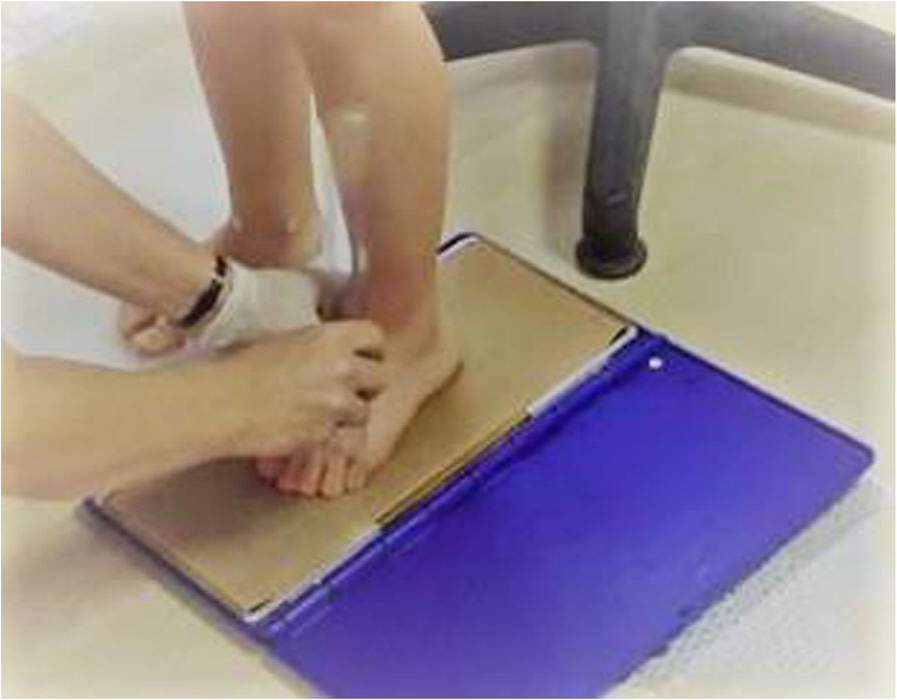
Pedigraph Methods

To study the footprint by pedigraph, three footprint measurements were used: Chippaux–Smirak, Arch Index and Staheli index. These parameters are usually used to categorize the footprint as pes cavus, pes planus or normal foot. The footprints were obtained by placing a reticulated piece of rubber sheeting, tensed and impregnated with ink, between the subject’s foot and a piece of stretched paper. In order to get an accurate footprint, it was performed while the participants were sitting and then knee was flexed while the footprint was taken weight-bearing in standing position (Fig. 1).

**Fig. 2:**
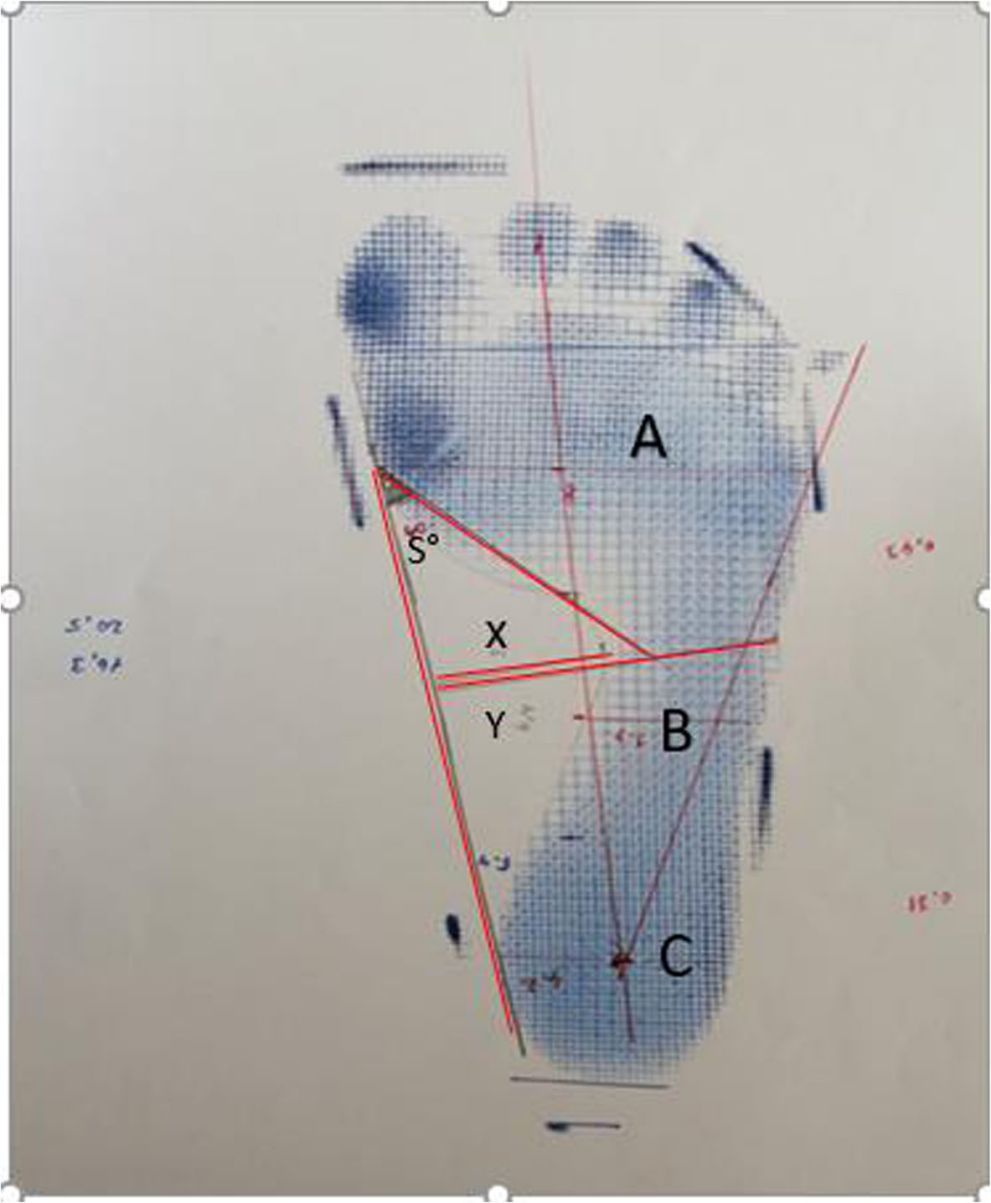
Footprint taken by pedigraph method

To determine the anthropometric dimensions of the foot, footprints were taken from the foot of the individuals using the pedigraph The TFL, FL, X (Arch width), Y (Midfoot width), S° (Clarks angle) [16], Arch Index (B / A + b + C), Chippaux Smirak Index (B / A * 100%) [17], Staheli Arc index [18], and foot rotation values of the children were examined (Fig. 2). (In addition, metatarsal circumference and oblique measurements were taken directly from the feet of children).

### Statistical analysis

Data were analyzed using the Statistical Package for Social Sciences (SPSS), version 23.0. When comparing the parameters, the normality values were first examined. Then, the independent samples t-test was used to analyze sex differences in terms of foot size and shape. To understand the relationship between other parameters, correlations analysis (Spearman correlation) was performed (*p* < 0.05).

**Fig. 3:**
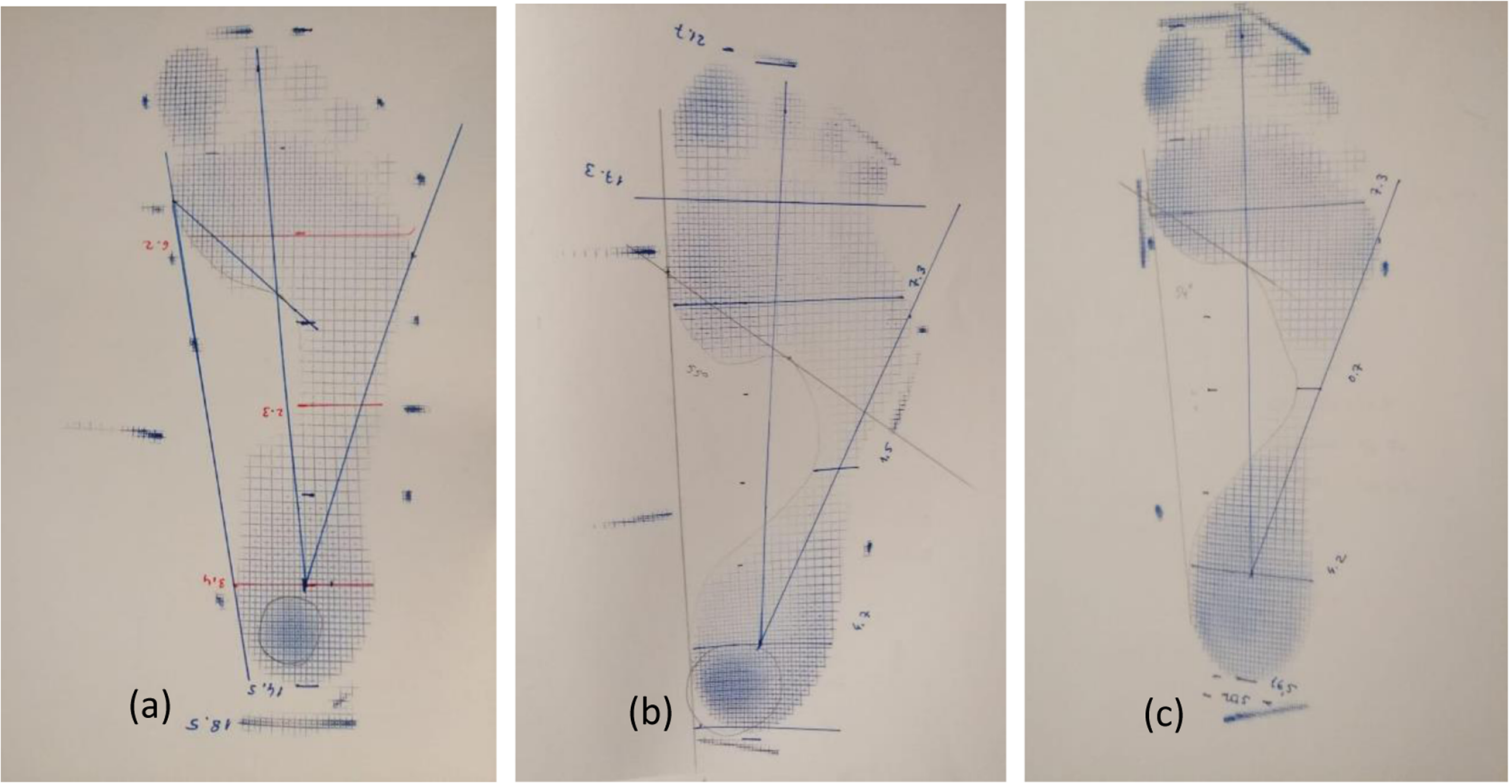
Arc height; (**a**) 1. ^o^ Pes Cavus, (**b**) 2. ^o^ Pes Cavus, (**c**) 3. ^o^ Pes Cavus

**Fig. 4:**
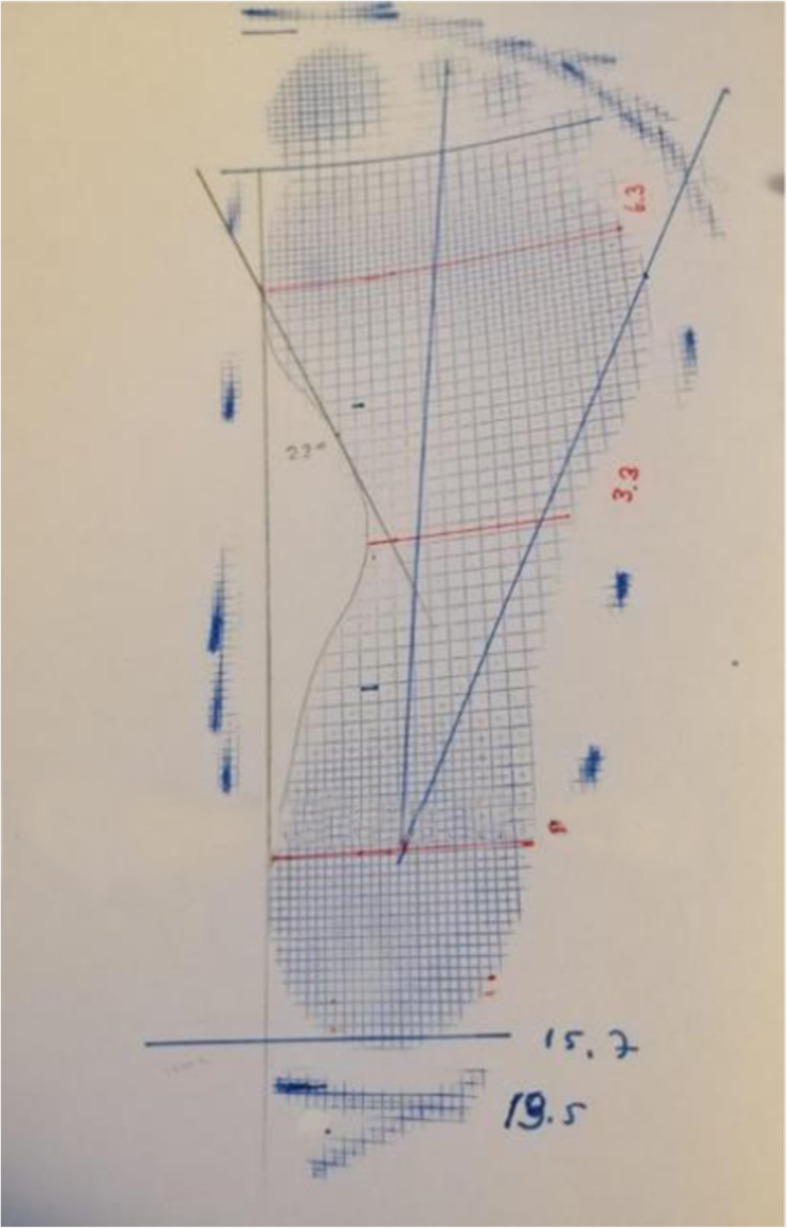
Normal arc structure

**Fig. 5:**
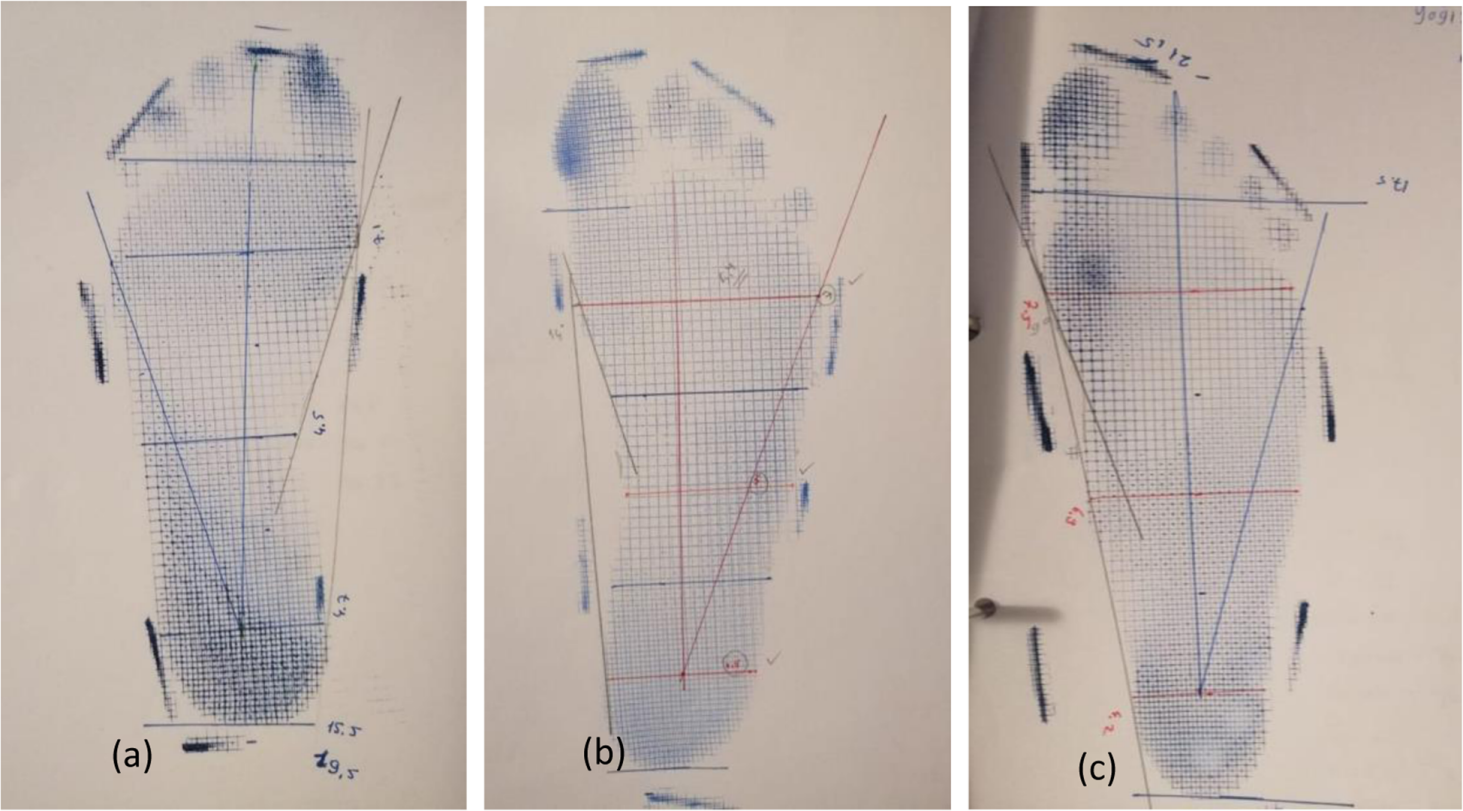
Arc drop; (**a**) 1. ^o^ Pes Planus, (**b**) 2. ^o^ Pes Planus, (**c**) 3. ^o^ Pes Planus

Staheli arc index was evaluated by forming seven different groups. The percentages of the participants were determined. Staheli arc index values between 0 and 19% were 3°; pes cavus, between 20 and 39% were 2°; pes cavus, between 40 and 59% were 1°; and pes cavus (Fig. 3), between 60 and 79% values as normal arc structure (Fig. 4), between 80 and 79% values as 1°, between 100 and 119% values as 2°, and 120% and above values as 3°. It was classified as pes planus and evaluated (Fig. 5).

## Results

The participants of the study consisted of 335 children (155 girls and 180 boys) aged between 6 and 10 (average: 8) years living in Central Anatolia. The body weights of the participants were between 14,60–55,50 kg and their heights were between 96 and 152 cm (Table 1).

**Table 1:** Demographic Data of Children

	Boys		Girls	
	Mean Value	SD	Mean Value	SD
Age (years)	7.48	1.4	7.59	1.3
Height (meter)	1.26	0.1	1.26	0.09
Body weight (kg)	28.35	7,8	27.93	7.4
Body Mass Index	17.35	2.5	17.30	2.7
Shoe Size	35.44	3.5	31.94	3.3

All descriptive statistics of the TFL (Truncated foot length), FL (foot length), Arch Index, Chippaux Smirak Index, Staheli Arc Index, X, Y, S°(Clarke angle), Metatarsal foot measurement (cm), Oblique foot measurement (cm) and foot rotation degree in boys and girl showed Tables 2 and 3. When the TFL values of the children were examined, the range was 11.50–19.80 cm, and the median was 15.5 cm. The normal distribution of the TFL values was found to be good. The range of the foot length was 14.12–24.89 cm, and the median was 19.28 cm. When the FL values were examined, the range was 14.00–25.50 cm, and the median was 19.2 cm; the values were found to be normally distributed. The range of the first metatarsal to ground distance was 0.83–3.80 cm, and the median value was 1.65 cm. When Chippaux Smirak index values were examined, the range was 0.00–1.01, and the median was 0.57.

**Table 2:** Data from Footprint Assessment

	Boys		Girls	
	Mean Value	SD	Mean Value	SD
Ark Endeks	11.37	1.1	11.25	0.8
Chippaux Smirak Index	0.62	0.1	0.56	0.1
Staheli Arc Index	%96.21	%27.86	%86.80	%26.10
X Value (cm)	1.56	1.06	1.96	1.04
Y Value (cm)	5.78	0.7	5.53	0.8
S Angle (^o^)	23.17	14.01	29.60	13.67
TFL (cm)	15.59	1.3	15.41	13,415
FL (cm)	19.45	1.7	19,2510	16,714
Foot Rotation (^o^)	21.96	2.6	21.38	2.28

**Table 3:** Data obtained from feet

	Boys		Girls	
	Mean Value	SD	Mean Value	SD
Metatarsal Measurement (cm)	18.46	1.6	18.04	1.4
Oblique (cm)	25.10	2.6	24.65	2.2

Further, the correlation (r = 0.177) between body mass index and foot rotation was found to be very weak.

When the correlation results of S value were analyzed, a weak correlation was observed between the S, Navicular ground distance and 1st Metatarsal ground distance. Additionally, a weak correlation (r = − 0.369) was found between the Chippaux Smirak index and Navicula to ground distance.

When the correlation analysis with other values of the Staheli Arc Index was examined, a weak correlation between the and navicula ground distance was observed.

A strong correlation (r = 0.795) was observed between the TFL and Metatarsal circumference, as well as between the TFL and oblique measurement (r = 0.848). Further, strong correlations were found between age and the TFL (r = 0.727), FL (r = 0.748), oblique measurement (r = 0.721), arc index (r = 0.717), shoe size (r = 0.723), and foot length (r = 0.650); however, the correlations between age and the 1st metatarsal to ground distance (r = 0.294), Chippaux Smirak Index (r = − 0.133), Staheli Arc Index (r = − 0.129), and foot rotation (r = − 0.030) were weak.

Foot length was strongly correlated with age (r = 0.736 for girls; r = 0.725 for boys). Foot length and height were strong correlated (r = 0.906 for girls; r = 0.887 for boys). Body weight and foot length of children were strongly correlated (r = 0.822 for girls; r = 0.836 for boys). There was a strong correlation between the metatarsal circumference measurements and foot length (r = 0.756 for girls; r = 0.817 for boys). The relationships between the oblique measure and foot height of children (r = 0.837 for girls; r = 0.858 for boys) and between the calculated arch index and foot length (r = 0.946 for girls; r = 0.946 for boys) were strong. It was determined that the foot height had a moderate relationship with the X angle (r = 0.695 for girls; r = 0.625 for boys). The distance of the medial malleolus to the ground was moderately related to the height of the foot (r = 0.526 for girls; r = 0.546 for boys).

A relationship was found between the metatarsal circumference and sex, which was stronger in the boys than in the girls (*p* = 0.18). No significant differences were observed between the sexes in terms of the 1st metatarsal to ground distance, TFL (*p* = 0.227), and foot length; however, there was a statistically significant difference in foot rotation between the sexes (*P* = 0.034), with girls having smaller foot rotation than boys.

According to the evaluation results with the classification made with the Staheli arch index, 0.30% of the sample was 3° pes cavus (1 individual [1 male]), 2.69% 2° pes cavus (9 individuals [1 male, 8 female]), 6.87% 1° pes cavus (23 individuals [9 boys, 14 girls]), 27.76% of the normal arch structure (93 individuals [48 boys, 45 girls]), 22.09% 1° pes planus (74 individuals [39 boys, 35 girls]), 17.70% 2° pes planus (66 individuals [37 boys, 29 girls]), and 20.60% 3° pes planus (69 individuals [45 boys, 24 girls]) were identified (Table 4).

**Table 4:** Arc classification distribution of children according to Staheli Arc Index

Values	Diagnosis	Number of Children	Boy	Girls
0–19%	High Arc (Pes Cavus) 3.^o^	1	1	0
20–39%	High Arc (Pes Cavus) 2.^o^	9	1	8
40–59%	High Arc (Pes Cavus) 1.^o^	23	9	14
60–79%	Normal Arc	93	48	45
80–99%	Low Arc (Pes Planus) 1.^o^	74	39	35
100–119%	Low Arc (Pes Planus) 2.^o^	66	37	29
120% and Over	Low Arc (Pes Planus) 3.^o^	69	45	24
	Total	335	180	155

According to the data determined in the classification made via the Staheli arch index, pes cavus is more common in girls than in boys. However, pes planus is more common in boys than in girls. Based on the Staheli arc index, almost the same number of girls and boys had a normal arc structure.

## Discussion

This study evaluated the foot measurements of children living in Central Anatolia using different methods. The rate of increase in foot sizes of girls and boys aged between 6 and 10 years varied. When the measurements of boys and girls of the same age were examined, it was found that the length of the TFL was smaller in boys than in girls. The results in this study demonstrate correlation between TFL, FL, oblique foot measurements, Chippaux Smirak index, Arch index, Staheli Arc Index and age in Anatolian children. We observed that Chippaux Smirak index, Arch index, Staheli Arc Index decreases as age increases. Previous studies in which the prevalence of pes planus decreased with increasing age [19–22].

Foot length was strongly correlated with age, body weight and height In addition to there was a strong correlation between the metatarsal circumference measurements, oblique measurements, the calculated arch index and foot length. The result of that investigation showed that increases in foot length due to anthropometric development in 6–10 year-old children. Leung, Cheng and Mak analysed the effect of age on foot length in 4–18 years old, and gradual change in the 6–12-year-old cohort. Müller et al. observed that foot length and width increased with age at rate similar to in the observed present study [21].

**Fig. 6:**
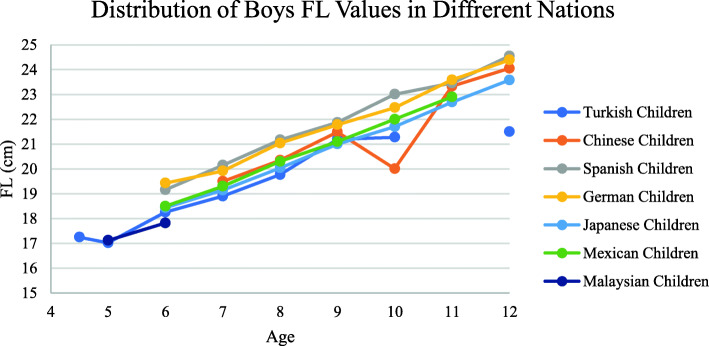
Distrubition of girls and boys FL values in different nations

In this study, FL values of Turkish children at different ages were examined. In this context, these were evaluated in different countries. Xu’s study also used data from Chinese children [23] . Delgado’s study included data of Spanish children [24]. Barisch’s [25] study included data of German children and Waseda’s [26] study included data from Japanese children. Data of Mexican children were included in Prado-Leon’s study [27]. In these studies, the age ranged from 6 to 12 years. In addition to the comparison, Bari’s study included boys aged 5 and 6 years [28]. The data are given in Fig. 6. According to this figure, we think that the foot length of Anatolian children may be closer to Asian children in terms of foot length.

**Fig. 7:**
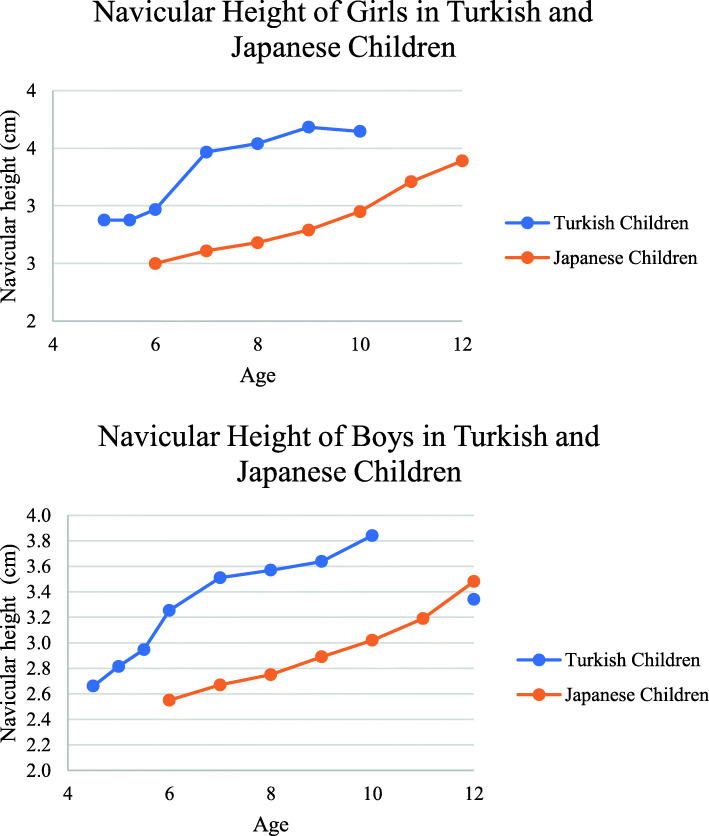
Navicular height of girls and boys in Turkish and Japanese children

The height of the navicular can be defined as the distance from the base of the medial projection of the navicular tuberosity to the ground. When the relationship between navicular height and age was examined, a low correlation was observed (r = 0.344 for girls; r = 0.354 for boys). Figure 7 shows the comparison of Turkish children with navicular height and Waseda’s (2014) study with Japanese children [26]. Some studies found that foot length was significantly associated with navicular height [29, 30]. Our study resulted that correlation between Chippaux Smirak index, Staheli Arc Index and navicular height.

**Fig. 8:**
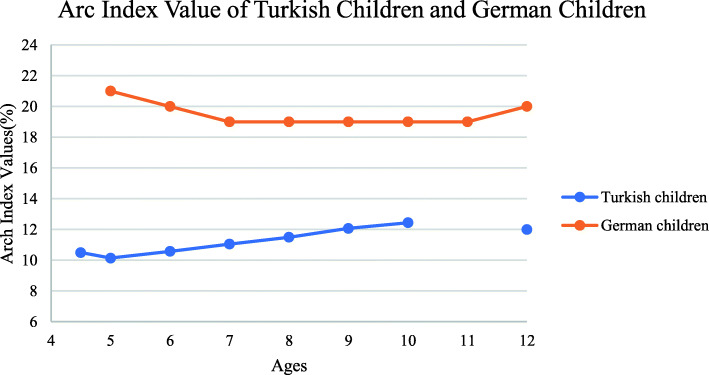
Arc index value of Turkish children and German children

The arc index value of Turkish children was also calculated and compared with that of children from other countries. In this context, the arch index values of German children were taken from Müller’s study [21]. The arch index values of children living in two different countries are given in Fig. 8. Hawes et al. and Shiang et al. also reported a significant cor relation between the arch index from inked footprint and the footprint index [31, 32].

**Fig. 9:**
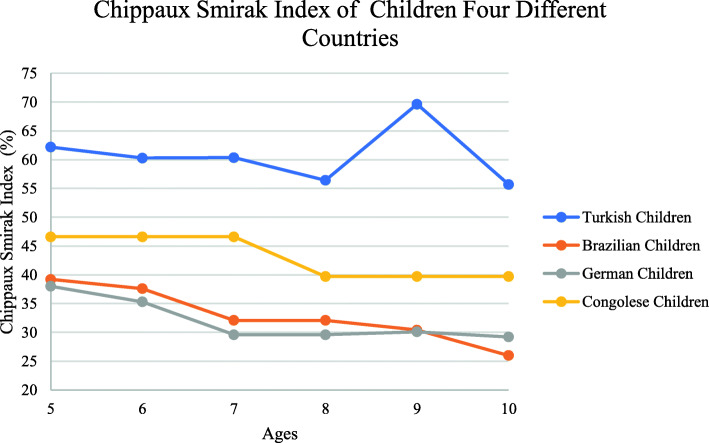
Chippaux Smirak index of children four different countries

The Chippaux Smirak index of Turkish children was examined by age [33]. Comparison of children with those from other nations has been made. Sacco’s [34] study obtained the values of Brazilian and German children. A comparison of Echarri’s [11] study with children in Congolese with Turkish children was made. The Chippaux Smirak indexes of children from four different countries are given in Fig. 9. Chippaux-Smirak index decreases up to age 11 in boys and 12 in girls [35]. This finding indicate that the physiological process of foot development from the low-arched types to normal types occurs, which is consistent with our result in age 10 year-old.

**Fig. 10:**
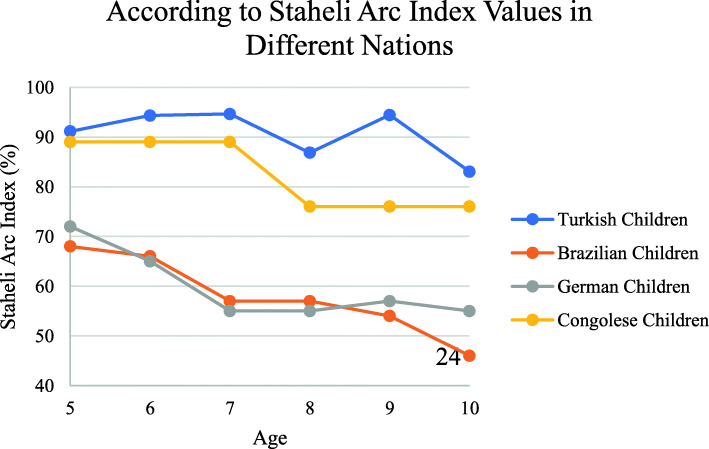
According to staheli arc index values in different nations

The Staheli Arc values of children were examined according to age [18]. However, the Staheli Arc values of Turkish children were compared with those of Brazilian, German, and Congolese children. Comparison of Staheli Arc values is given in Fig. 10. In the present study, the frequency of low ach type feet found in the youngest age groups decreased with age by Staheli index and Chippaux Smirak index, this is in accordance with earlier studies [33, 36]. In this study found that 62,3% pes planus, 9.8% pes cavus and 27.7% of the normal arch structure by classification made with the Staheli arch index. Ibeabuchi et al. showed that the overall percentage of subjects with flatfeet was 59 (13.53%) out of a total number of 436 ft analysed in 6–15-year-old Nigerian school children by classification made with the Staheli arch index [37].

Our results that foot rotation increased with body mass index in the girls compared to that in the boys. The gradual ossification of bony structures may lead to better stabilization of the arch during weight bearing [38]. This result regarding that the ongoing external tibial rotation, from the in-toeing position after birth to the out-toeing position during growth, results in proportional decrease in the valgus configuration of the hindfoot and elevation of the MLA [39].

The data of the study varies according to the number of individuals participating in the sampling and the techniques used to measure the foot structure; therefore, increasing the sample size and the use of three-dimensional scanning system to analyze Central Anatolia different regions of the foot and arch structure is proposed.

## Conclusion

This study aimed to determine the foot sizes and foot structures of children living in Central Anatolia. Foot structures of children aged 6–10 years were shown to be different according to sex and increasing age. Age, gender and body weight were associated with foot dimensions and pes planus diagnosis. According to classification made with the Staheli arch index, 63.3% pes planus, 9.8% pes cavus and 27.7% of the normal arch structure were identified in Anatolian child. The results of the study may be used as an important resource for shoe designers in terms of child foot health.

## Data Availability

All the data that support the findings of this study are contained within the manuscript. Any requests for additional data can be made available upon reasonable request from the corresponding author and with permission of Ethics Department of the University of Ankara.
